# 2-Bromo-4-chloro-6-{(*E*)-[4-(diethyl­amino)­phen­yl]imino­meth­yl}phenol

**DOI:** 10.1107/S1600536810033738

**Published:** 2010-08-28

**Authors:** K. Manvizhi, S. Ranjith, K. Parthiban, G. Rajagopal, A. SubbiahPandi

**Affiliations:** a Department of Chemistry, Anand Institute of Higher Technology, Kazhipattur, Chennai 603 103, India; b Department of Physics, Presidency College (Autonomous), Chennai 600 005, India; c Department of Chemistry, Pondicherry University, Pondicherry 605 014, India; d Department of Chemistry, Government Arts College, Melur 625 106, India

## Abstract

In the title compound, C_17_H_18_BrClN_2_O, the dihedral angle between the aromatic rings is 3.0 (1)°. The methyl­ethanamine group assumes an extended conformation. An intra­molecular O—H⋯N hydrogen bond generates an *S*(6) ring motif. The crystal packing is stabilized by C—H⋯π and π–π [centroid–centroid distances = 3.691 (1) and 3.632 (1) Å] inter­actions.

## Related literature

For Schiff base compounds in coordination chemistry, see: Weber *et al.* (2007 ▶); Chen *et al.* (2008 ▶) and for their role in biological processes, see: May *et al.* (2004 ▶). For hydrogen-bond motifs, see: Bernstein *et al.* (1995 ▶). For related structures, see: Raja *et al.* (2008 ▶).
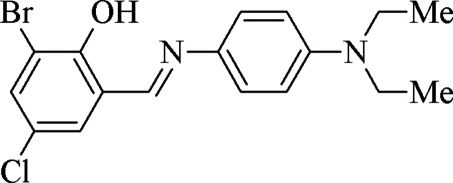


## Experimental

### 

#### Crystal data

C_17_H_18_BrClN_2_O
*M* *_r_* = 381.69
Monoclinic, 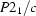

*a* = 11.3427 (3) Å
*b* = 10.9204 (3) Å
*c* = 14.3869 (4) Å
β = 111.418 (2)°
*V* = 1658.99 (8) Å^3^
*Z* = 4
Mo *K*α radiation
μ = 2.64 mm^−1^
*T* = 293 K
0.21 × 0.19 × 0.17 mm

#### Data collection

Bruker Kappa APEXII CCD diffractometer
Absorption correction: multi-scan (*SADABS*; Sheldrick, 1996 ▶) *T*_min_ = 0.972, *T*_max_ = 0.977
19985 measured reflections
4383 independent reflections
2797 reflections with *I* > 2σ(*I*)
*R*_int_ = 0.034

#### Refinement

*R*[*F* ^2^ > 2σ(*F* ^2^)] = 0.034
*wR*(*F* ^2^) = 0.088
*S* = 1.00
4383 reflections
202 parameters
H-atom parameters constrained
Δρ_max_ = 0.27 e Å^−3^
Δρ_min_ = −0.28 e Å^−3^

### 

Data collection: *APEX2* (Bruker, 2004 ▶); cell refinement: *SAINT* (Bruker, 2004 ▶); data reduction: *SAINT*; program(s) used to solve structure: *SHELXS97* (Sheldrick, 2008 ▶); program(s) used to refine structure: *SHELXL97* (Sheldrick, 2008 ▶); molecular graphics: *ORTEP-3* (Farrugia, 1997 ▶); software used to prepare material for publication: *SHELXL97* and *PLATON* (Spek, 2009 ▶).

## Supplementary Material

Crystal structure: contains datablocks global, I. DOI: 10.1107/S1600536810033738/gw2086sup1.cif

Structure factors: contains datablocks I. DOI: 10.1107/S1600536810033738/gw2086Isup2.hkl

Additional supplementary materials:  crystallographic information; 3D view; checkCIF report

## Figures and Tables

**Table 1:** Hydrogen-bond geometry (Å, °) *Cg*2 is the centroid of the C8–C13 ring.

*D*—H⋯*A*	*D*—H	H⋯*A*	*D*⋯*A*	*D*—H⋯*A*
O1—H1*A*⋯N1	0.82	1.86	2.588 (2)	147
C16—H16*A*⋯*Cg*2^i^	0.96	2.90	3.814 (2)	157

Symmetry code: (i) 
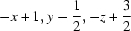
.

**Fig. 1.**
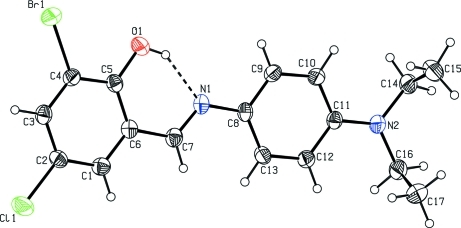
The molecular structure of the title compound showing 30% probability displacement ellipsoids. The intramolecular O–H···N interaction is shown as dashed lines

**Fig. 2.**
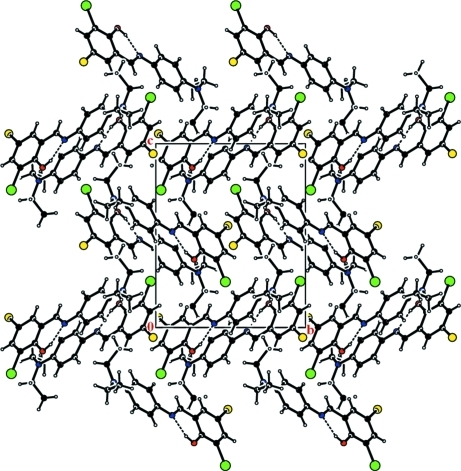
The packing diagram of the title compound, view along the a axis.

## supplementary crystallographic information

### Comment

The Schiff base compounds have received considerable attention for many years, primarily due to their importance in the development of coordination chemistry related to magnetism (Weber *et al.*, 2007), catalysis (Chen *et al.*, 2008) and biological process (May *et al.*, 2004). Against this background, and in order to obtain detailed information on molecular conformations in the solid state, X-ray studies of the title compound have been carried out.

X-Ray analysis confirms the molecular structure and atom connectivity as illustrated in Fig. 1. The geometric parameters of the title molecule agrees well with those reported for a similar structure (Raja *et al.*, 2008). The methylethanamine moiety assumes an extended conformation as can be seen from torsion angles C15–C14–N2–C11 of 76.5 (2)° and C17–C16–N2–C11 of -74.1 (2)°. The atoms Cl1, Br1 and O1 are deviated by -0.039 (1), 0.009 (1) and -0.040 (2)Å from the least square plane of the ring C1–C6 and also atoms N1 and N2 are deviated by 0.016 (2) and -0.029 (2)Å from the least square plane of the ring C8–C13. The dihedral angle between the aromatic rings is 3.0 (1)°, shows that both the rings(C1–C6 and C8–C13) are almost coplanar.

In addition to the van der Waals interactions, the crystal packing is stabilized by O–H···N and C–H···π hydrogen bonds (Table. 1) as well as by π–π electron interaction. The intramolecular O–H···N hydrogen bond which generates an S(6) ring motif (Fig.1) (Bernstein *et al.*, 1995). The π–π electron interactions between the rings *Cg*1···*Cg*1 and *Cg*1···*Cg*2 at -*x*, 1 - *y*, 1 - *z* and -*x*, -*y*, 1 - *z* with the centroid–centroid distance equal to 3.691 (1) and 3.632 (1) Å, respectively are observed in the crystal structure [*Cg*1 and *Cg*2 are the centroids of the rings C1–C6 and C8–C13].

### Experimental

An ethanoic solution (20 ml) *N*,*N*-Diethyl aniline (10 mmol) was magnetically stirred in a round bottom flask followed by dropwise addition of Bromo- Chloro Salicylaldehyde (10 mmol). The reaction mixture was then refluxed for three hours and upon cooling to 0°C an red crystalline solid precipitates from the mixture. The solid which is separated out was filtered washed with ice cold ethanol and dried in vaccuo over anhydrous CaCl_2_. Single crystals suitable for the X-ray diffraction were obtained by slow evaporation of a solution of the title compound in ethyl acetate at room temperature.

### Refinement

All the H atoms were positioned geometrically, with O—H = 0.82 Å and C—H = 0.93 - 0.98 Å and constrained to ride on their parent atom, with *U*_iso_H=1.2*U*_eq_(C).

### Crystal data

**Table:**

C_17_H_18_BrClN_2_O	*F*(000) = 776
*M**_r_* = 381.69	*D*_x_ = 1.528 Mg m^−^^3^
Monoclinic, *P*2_1_/*c*	Mo *K*α radiation, λ = 0.71073 Å
Hall symbol: -P 2ybc	Cell parameters from 4383 reflections
*a* = 11.3427 (3) Å	θ = 1.9–28.9°
*b* = 10.9204 (3) Å	µ = 2.64 mm^−^^1^
*c* = 14.3869 (4) Å	*T* = 293 K
β = 111.418 (2)°	Block, colourless
*V* = 1658.99 (8) Å^3^	0.21 × 0.19 × 0.17 mm
*Z* = 4	

### Data collection

**Table:**

Bruker Kappa APEXII CCD diffractometer	4383 independent reflections
Radiation source: fine-focus sealed tube	2797 reflections with *I* > 2σ(*I*)
graphite	*R*_int_ = 0.034
ω scans	θ_max_ = 28.9°, θ_min_ = 1.9°
Absorption correction: multi-scan (*SADABS*; Sheldrick, 1996)	*h* = −14→15
*T*_min_ = 0.972, *T*_max_ = 0.977	*k* = −14→14
19985 measured reflections	*l* = −18→19

### Refinement

**Table:**

Refinement on *F*^2^	Primary atom site location: structure-invariant direct methods
Least-squares matrix: full	Secondary atom site location: difference Fourier map
*R*[*F*^2^ > 2σ(*F*^2^)] = 0.034	Hydrogen site location: inferred from neighbouring sites
*wR*(*F*^2^) = 0.088	H-atom parameters constrained
*S* = 1.00	*w* = 1/[σ^2^(*F*_o_^2^) + (0.0389*P*)^2^ + 0.3262*P*] where *P* = (*F*_o_^2^ + 2*F*_c_^2^)/3
4383 reflections	(Δ/σ)_max_ < 0.001
202 parameters	Δρ_max_ = 0.27 e Å^−^^3^
0 restraints	Δρ_min_ = −0.28 e Å^−^^3^

### Special details

**Table:**

Geometry. All e.s.d.'s (except the e.s.d. in the dihedral angle between two l.s. planes) are estimated using the full covariance matrix. The cell e.s.d.'s are taken into account individually in the estimation of e.s.d.'s in distances, angles and torsion angles; correlations between e.s.d.'s in cell parameters are only used when they are defined by crystal symmetry. An approximate (isotropic) treatment of cell e.s.d.'s is used for estimating e.s.d.'s involving l.s. planes.
Refinement. Refinement of *F*^2^ against ALL reflections. The weighted *R*-factor *wR* and goodness of fit *S* are based on *F*^2^, conventional *R*-factors *R* are based on *F*, with *F* set to zero for negative *F*^2^. The threshold expression of *F*^2^ > σ(*F*^2^) is used only for calculating *R*-factors(gt) *etc*. and is not relevant to the choice of reflections for refinement. *R*-factors based on *F*^2^ are statistically about twice as large as those based on *F*, and *R*- factors based on ALL data will be even larger.

### Fractional atomic coordinates and isotropic or equivalent isotropic displacement parameters (Å^2^)

**Table:**

	*x*	*y*	*z*	*U*_iso_*/*U*_eq_	
C1	−0.10155 (19)	0.32018 (18)	0.54970 (15)	0.0458 (5)	
H1	−0.1023	0.2923	0.6106	0.055*	
C2	−0.17812 (19)	0.41572 (19)	0.50175 (15)	0.0443 (5)	
C3	−0.17718 (19)	0.45989 (18)	0.41258 (15)	0.0456 (5)	
H3	−0.2285	0.5256	0.3812	0.055*	
C4	−0.09947 (18)	0.40567 (19)	0.37050 (14)	0.0434 (5)	
C5	−0.02201 (17)	0.30769 (18)	0.41549 (14)	0.0412 (4)	
C6	−0.02323 (17)	0.26536 (17)	0.50738 (14)	0.0398 (4)	
C7	0.05636 (18)	0.16390 (18)	0.55862 (15)	0.0450 (5)	
H7	0.0544	0.1382	0.6197	0.054*	
C8	0.20773 (17)	0.01035 (17)	0.57157 (15)	0.0393 (4)	
C9	0.28861 (19)	−0.03587 (18)	0.52790 (14)	0.0443 (5)	
H9	0.2875	−0.0019	0.4683	0.053*	
C10	0.37053 (19)	−0.13055 (19)	0.56995 (14)	0.0457 (5)	
H10	0.4238	−0.1589	0.5385	0.055*	
C11	0.37521 (17)	−0.18518 (17)	0.65933 (14)	0.0393 (4)	
C12	0.29288 (18)	−0.13766 (18)	0.70294 (15)	0.0446 (5)	
H12	0.2933	−0.1712	0.7625	0.054*	
C13	0.21127 (18)	−0.04266 (18)	0.66006 (16)	0.0441 (5)	
H13	0.1577	−0.0136	0.6910	0.053*	
C14	0.5476 (2)	−0.3221 (2)	0.66057 (16)	0.0530 (5)	
H14A	0.5074	−0.3263	0.5885	0.064*	
H14B	0.5745	−0.4042	0.6846	0.064*	
C15	0.6624 (2)	−0.2420 (3)	0.6864 (2)	0.0701 (7)	
H15A	0.6367	−0.1591	0.6672	0.105*	
H15B	0.7150	−0.2701	0.6515	0.105*	
H15C	0.7091	−0.2454	0.7570	0.105*	
C16	0.4686 (2)	−0.3271 (2)	0.79959 (16)	0.0576 (6)	
H16A	0.5081	−0.4072	0.8084	0.069*	
H16B	0.3851	−0.3373	0.8026	0.069*	
C17	0.5454 (2)	−0.2467 (3)	0.88435 (17)	0.0756 (8)	
H17A	0.6295	−0.2386	0.8840	0.113*	
H17B	0.5494	−0.2826	0.9463	0.113*	
H17C	0.5066	−0.1674	0.8771	0.113*	
N1	0.12906 (14)	0.10877 (15)	0.52225 (12)	0.0432 (4)	
N2	0.45430 (15)	−0.28149 (15)	0.70127 (12)	0.0462 (4)	
Cl1	−0.27823 (7)	0.48188 (6)	0.55421 (5)	0.07030 (19)	
Br1	−0.09834 (3)	0.46511 (3)	0.248320 (18)	0.07298 (12)	
O1	0.04963 (14)	0.25578 (15)	0.37088 (11)	0.0584 (4)	
H1A	0.0942	0.2028	0.4074	0.088*	

### Atomic displacement parameters (Å^2^)

**Table:**

	*U*^11^	*U*^22^	*U*^33^	*U*^12^	*U*^13^	*U*^23^
C1	0.0515 (12)	0.0407 (11)	0.0471 (11)	−0.0004 (9)	0.0205 (9)	0.0027 (9)
C2	0.0473 (11)	0.0392 (11)	0.0513 (12)	0.0029 (9)	0.0238 (9)	−0.0033 (9)
C3	0.0467 (11)	0.0368 (11)	0.0504 (12)	0.0064 (9)	0.0144 (9)	0.0023 (9)
C4	0.0454 (11)	0.0435 (12)	0.0404 (10)	0.0014 (9)	0.0146 (9)	0.0028 (9)
C5	0.0361 (10)	0.0411 (11)	0.0455 (11)	−0.0008 (8)	0.0137 (8)	−0.0042 (9)
C6	0.0382 (10)	0.0325 (10)	0.0465 (11)	−0.0027 (8)	0.0127 (8)	0.0002 (9)
C7	0.0430 (11)	0.0403 (11)	0.0498 (11)	0.0003 (9)	0.0146 (9)	0.0063 (9)
C8	0.0317 (9)	0.0362 (10)	0.0446 (10)	−0.0018 (8)	0.0073 (8)	−0.0015 (8)
C9	0.0468 (11)	0.0466 (12)	0.0365 (9)	0.0018 (9)	0.0118 (8)	0.0019 (9)
C10	0.0487 (11)	0.0493 (13)	0.0397 (10)	0.0092 (9)	0.0166 (9)	−0.0005 (9)
C11	0.0366 (10)	0.0368 (10)	0.0398 (10)	0.0008 (8)	0.0082 (8)	−0.0027 (8)
C12	0.0438 (11)	0.0461 (12)	0.0444 (10)	0.0017 (9)	0.0167 (9)	0.0060 (9)
C13	0.0384 (10)	0.0451 (12)	0.0515 (11)	0.0040 (9)	0.0195 (9)	0.0028 (10)
C14	0.0579 (13)	0.0475 (13)	0.0525 (12)	0.0171 (10)	0.0189 (10)	0.0001 (10)
C15	0.0561 (14)	0.0846 (19)	0.0759 (16)	0.0073 (13)	0.0316 (13)	−0.0034 (15)
C16	0.0572 (14)	0.0560 (14)	0.0612 (14)	0.0143 (11)	0.0236 (11)	0.0179 (12)
C17	0.0747 (17)	0.103 (2)	0.0468 (13)	0.0149 (16)	0.0191 (12)	0.0031 (14)
N1	0.0349 (8)	0.0399 (9)	0.0490 (9)	0.0008 (7)	0.0085 (7)	0.0018 (8)
N2	0.0459 (9)	0.0451 (10)	0.0462 (9)	0.0102 (8)	0.0152 (8)	0.0054 (8)
Cl1	0.0855 (4)	0.0679 (4)	0.0736 (4)	0.0250 (3)	0.0482 (4)	0.0040 (3)
Br1	0.0821 (2)	0.0911 (2)	0.05317 (15)	0.02883 (15)	0.03355 (13)	0.02548 (13)
O1	0.0581 (9)	0.0646 (11)	0.0610 (9)	0.0203 (8)	0.0318 (8)	0.0094 (8)

### Geometric parameters (Å, °)

**Table:**

C1—C2	1.372 (3)	C11—N2	1.371 (2)
C1—C6	1.383 (3)	C11—C12	1.401 (3)
C1—H1	0.9300	C12—C13	1.377 (3)
C2—C3	1.374 (3)	C12—H12	0.9300
C2—Cl1	1.734 (2)	C13—H13	0.9300
C3—C4	1.373 (3)	C14—N2	1.453 (3)
C3—H3	0.9300	C14—C15	1.499 (3)
C4—C5	1.386 (3)	C14—H14A	0.9700
C4—Br1	1.878 (2)	C14—H14B	0.9700
C5—O1	1.332 (2)	C15—H15A	0.9600
C5—C6	1.405 (3)	C15—H15B	0.9600
C6—C7	1.449 (3)	C15—H15C	0.9600
C7—N1	1.277 (3)	C16—N2	1.452 (3)
C7—H7	0.9300	C16—C17	1.497 (3)
C8—C9	1.383 (3)	C16—H16A	0.9700
C8—C13	1.386 (3)	C16—H16B	0.9700
C8—N1	1.412 (2)	C17—H17A	0.9600
C9—C10	1.374 (3)	C17—H17B	0.9600
C9—H9	0.9300	C17—H17C	0.9600
C10—C11	1.401 (3)	O1—H1A	0.8200
C10—H10	0.9300		
			
C2—C1—C6	119.89 (19)	C13—C12—H12	119.1
C2—C1—H1	120.1	C11—C12—H12	119.1
C6—C1—H1	120.1	C12—C13—C8	121.03 (19)
C1—C2—C3	121.14 (19)	C12—C13—H13	119.5
C1—C2—Cl1	119.54 (16)	C8—C13—H13	119.5
C3—C2—Cl1	119.32 (16)	N2—C14—C15	114.65 (19)
C4—C3—C2	119.05 (18)	N2—C14—H14A	108.6
C4—C3—H3	120.5	C15—C14—H14A	108.6
C2—C3—H3	120.5	N2—C14—H14B	108.6
C3—C4—C5	121.78 (19)	C15—C14—H14B	108.6
C3—C4—Br1	119.25 (15)	H14A—C14—H14B	107.6
C5—C4—Br1	118.96 (15)	C14—C15—H15A	109.5
O1—C5—C4	119.85 (18)	C14—C15—H15B	109.5
O1—C5—C6	122.04 (17)	H15A—C15—H15B	109.5
C4—C5—C6	118.11 (18)	C14—C15—H15C	109.5
C1—C6—C5	120.02 (18)	H15A—C15—H15C	109.5
C1—C6—C7	119.20 (18)	H15B—C15—H15C	109.5
C5—C6—C7	120.78 (18)	N2—C16—C17	114.7 (2)
N1—C7—C6	121.83 (19)	N2—C16—H16A	108.6
N1—C7—H7	119.1	C17—C16—H16A	108.6
C6—C7—H7	119.1	N2—C16—H16B	108.6
C9—C8—C13	117.61 (18)	C17—C16—H16B	108.6
C9—C8—N1	116.88 (18)	H16A—C16—H16B	107.6
C13—C8—N1	125.51 (19)	C16—C17—H17A	109.5
C10—C9—C8	121.93 (19)	C16—C17—H17B	109.5
C10—C9—H9	119.0	H17A—C17—H17B	109.5
C8—C9—H9	119.0	C16—C17—H17C	109.5
C9—C10—C11	121.17 (19)	H17A—C17—H17C	109.5
C9—C10—H10	119.4	H17B—C17—H17C	109.5
C11—C10—H10	119.4	C7—N1—C8	122.49 (18)
N2—C11—C12	121.54 (18)	C11—N2—C16	120.96 (17)
N2—C11—C10	122.01 (18)	C11—N2—C14	120.81 (17)
C12—C11—C10	116.44 (17)	C16—N2—C14	116.70 (16)
C13—C12—C11	121.82 (19)	C5—O1—H1A	109.5
			
C6—C1—C2—C3	−1.1 (3)	C8—C9—C10—C11	0.3 (3)
C6—C1—C2—Cl1	178.74 (15)	C9—C10—C11—N2	178.53 (18)
C1—C2—C3—C4	1.0 (3)	C9—C10—C11—C12	−0.3 (3)
Cl1—C2—C3—C4	−178.80 (15)	N2—C11—C12—C13	−178.59 (18)
C2—C3—C4—C5	0.0 (3)	C10—C11—C12—C13	0.3 (3)
C2—C3—C4—Br1	179.70 (15)	C11—C12—C13—C8	−0.2 (3)
C3—C4—C5—O1	178.33 (19)	C9—C8—C13—C12	0.2 (3)
Br1—C4—C5—O1	−1.4 (3)	N1—C8—C13—C12	−179.17 (18)
C3—C4—C5—C6	−0.9 (3)	C6—C7—N1—C8	179.57 (17)
Br1—C4—C5—C6	179.36 (14)	C9—C8—N1—C7	−175.52 (18)
C2—C1—C6—C5	0.1 (3)	C13—C8—N1—C7	3.9 (3)
C2—C1—C6—C7	−179.53 (18)	C12—C11—N2—C16	−8.6 (3)
O1—C5—C6—C1	−178.37 (18)	C10—C11—N2—C16	172.58 (19)
C4—C5—C6—C1	0.9 (3)	C12—C11—N2—C14	−174.04 (18)
O1—C5—C6—C7	1.3 (3)	C10—C11—N2—C14	7.2 (3)
C4—C5—C6—C7	−179.49 (18)	C17—C16—N2—C11	−74.1 (2)
C1—C6—C7—N1	178.95 (18)	C17—C16—N2—C14	91.9 (2)
C5—C6—C7—N1	−0.7 (3)	C15—C14—N2—C11	76.5 (2)
C13—C8—C9—C10	−0.3 (3)	C15—C14—N2—C16	−89.5 (2)
N1—C8—C9—C10	179.17 (18)		

### Hydrogen-bond geometry (Å, °)

**Table:**

Cg2 is the centroid of the C8–C13 ring.

**Table:**

*D*—H···*A*	*D*—H	H···*A*	*D*···*A*	*D*—H···*A*
O1—H1A···N1	0.82	1.86	2.588 (2)	147
C16—H16A···Cg2^i^	0.96	2.90	3.814 (2)	157

Symmetry codes:  (i) −*x*+1, *y*−1/2, −*z*+3/2.
